# Nanomaterial-mediated low-temperature photothermal therapy *via* heat shock protein inhibition

**DOI:** 10.3389/fbioe.2022.1027468

**Published:** 2022-10-11

**Authors:** Yu Xin, Zhuokai Sun, Jie Liu, Wei Li, Meirong Wang, Yongli Chu, Zhihong Sun, Guanjun Deng

**Affiliations:** ^1^ Yantai Yuhuangding Hospital, Yantai, China; ^2^ Nanchang University Queen Mary School, Nanchang, China; ^3^ School of Pharmaceutical Sciences (Shenzhen), Sun Yat-Sen University, Shenzhen, China

**Keywords:** low-temperature, photothermal therapy, heat shock proteins, nanotechnology, nanomaterial

## Abstract

With the continuous development of nanobiotechnology in recent years, combining photothermal materials with nanotechnology for tumor photothermal therapy (PTT) has drawn many attentions nanomedicine research. Although nanomaterial-mediated PTT is more specific and targeted than traditional treatment modalities, hyperthermia can also damage normal cells. Therefore, researchers have proposed the concept of low-temperature PTT, in which the expression of heat shock proteins (HSPs) is inhibited. In this article, the research strategies proposed in recent years based on the inhibition of HSPs expression to achieve low-temperature PTT was reviewed. Folowing this, the synthesis, properties, and applications of these nanomaterials were introduced. In addition, we also summarized the problems of nanomaterial-mediated low-temperature PTT at this stage and provided an outlook on future research directions.

## 1 Introduction

Traditional treatments for tumors mainly include surgery, radiotherapy (RT), and chemotherapy. However, these treatments, may have problems such as incomplete treatment and many associated side effects. Researchers have been seeking more targeted and effective treatment methods due to high morbidity and mortality. In recent years, with the rapid development of synthetic technology, the new therapeutic modality of photothermal therapy (PTT) has been recognized as one of the most promising treatments to attract widespread attention in the biomedical field (Pinto and Pocard, 2018; Liu et al., 2019; Li X. et al., 2020; Lu et al., 2021; Lv et al., 2021; Peng et al., 2021; Zafar et al., 2021). The main treatment principle of PTT is to use photosensitizers (such as indocyanine green (ICG), *etc.*) that absorb in the near-infrared (NIR) to produce thermal therapeutic effects for tumor elimination. Compared with traditional anti-tumor therapies (such as RT and chemotherapy), photosensitizers are non-toxic substances in the dark, which makes NIR-triggered PTT more significantly reduce toxic side effects; in addition, the light radiation parameters have great specificity (such as duration, power intensity and location), can be precisely controlled, which further increases its therapeutic effect. For sustainable efficient photothermal systems, researchers adopted black nanomaterials as light absorber to boost the absorption and conversion efficiency of photothermal materials (Liu et al., 2017; Wang L. et al., 2021). Moreover, many materials exhibit high absorption peaks in the NIR band and can be used as effective photothermal sensors (Pelaz et al., 2017; Fernandes et al., 2020; Lee and Gaharwar, 2020; Zhang et al., 2020; Wang Y. et al., 2021; Yu et al., 2021). Furthermore, changes in synthesis methods of materials can modulate the light absorption peaks to a narrow wavelength range, improving the specificity of light absorption;

For the conventional PTT treatment, photothermal agent with strong photothermal conversion efficiency was mainly synthesized through various assembly modifications. After injecting into the body, the photothermal agent accumulates at the tumor site, and transform the light to heat conversion can kill tumor cells under external irradiation (e.g., NIR laser) (Melancon et al., 2011; Zhou et al., 2016; De Melo-Diogo et al., 2017). However, PTT has some disadvantages, which mainly disadvantages were heat-shock response and limited light penetration. As previous studies shown, the temperature >45°C is usually required to achieve the photothermal damage effect on tumor cells. Based on this, a higher temperature (>50°C) is usually required in the centre of the tumor site and then gradually a temperature gradient difference forms outward so that the marginal parts of the tumor also reach the treatment temperature (Tang, 2011; Liu et al., 2019; Sun Q. et al., 2021). Beside this, the limited light penetration is also the focus issue for PTT clinical application.

Fueled by the improving nanotechnology, the application of nanomaterials in PTT is one of the hotspots in nanomedicine research. Nanomaterials are one-dimensional materials with sizes between 1 and 100 nm and have a variety of unique properties that depend on their chemical structure, synthesis methods and modifications. Nanomaterial-based PTT has many advantages in tumor therapy. Many nanomaterials can be attached to various surface modification molecules (e.g. polymers or antibodies), which can alter the biological properties of the nanomaterials and attenuate or eliminate the toxicity of some nanomaterials (Guo and Wang, 2011; Doughty et al., 2019; Obuobi et al., 2019; Andraos and Gulumian, 2021; Subjakova et al., 2021). Firstly, the highly specific and target to tumor site which could reduce damage to normal tissue cells. Furthermore, adjustable effects on the tumor site can be produced *via* adjusting the NIR laser irradiation parameters (e.g., time, power, and site). In addition, Nanomaterial-based PTT can promote photothermal agents selective accumulation in tumors *via* enhancing permeability and retention (EPR) effects, which significantly improved both the selectivity and efficacy of PTT and have been used to treat many types of tumors. However, under the stimulation of high temperature, although PTT will have a better killing effect on tumor cells, it may also produce certain damage to the surrounding healthy tissues and cells (Zhu et al., 2016); For example, the cell damage caused by high temperature will release some intracellular factors, which may aggravate local tissue inflammation (Bonfil et al., 1988). Meanwhile, tumor cells stimulated by high temperature easily acquire tolerance to hyperthermia, which will further enhance the viability of tumor cells, a phenomenon known as thermotolerance (Kampinga, 1993; Wu, 1995). In response to the many problems associated with high-temperature PTT, the research strategy of low-temperature PTT has been proposed in recent years (Hu et al., 2021; Yi et al., 2021).

Unlike high power laser irradiation in high temperature PTT, low-temperature PTT mainly refers to the induction of tumor cell apoptosis under low power laser irradiation, i.e. low temperature (≤45°C) photothermal conditions (Jung et al., 2016; Xia et al., 2021). Although low-temperature PTT reduces the damage to normal tissue cells, it may not be able to achieve a satisfactory anti-tumor therapeutic effect due to the existence of molecular chaperones and heat shock proteins (HSPs) that are associated with heat resistance in living organisms. HSPs are highly conserved and present in almost all cells and perform various concomitant functions, which involved in nascent polypeptide chain formation, protein folding, protein transport and antigen delivery (Morimoto, 1998). HSPs are located inside and outside normal cells but also in tumor cells and virus/bacterial-infected cells and facilitate resistance to adverse environmental factors (e.g. high temperature or toxic environment stimulation) (Chen et al., 1999; Sherman and Multhoff, 2007; Liang et al., 2015). HSPs are widely expressed in tumor cells and play an important role in mediating intrinsic tumor cell characteristics, such as regulating non-programmed cell division, evading programmed cell death and senescence, promoting neovascularization and increasing metastasis and invasion (Li et al., 1995). Moreover, HSPs take part in the interaction between tumor cells and the TME, which can promote the release of HSPs from tumor cells to influence the behaviour of neighboring cells and infiltrate normal cell (Lang et al., 2019; Marchan, 2019; Wang et al., 2020; Seclì et al., 2021; Fu and Jia, 2022).

Therefore, in the application of low-temperature PTT, it is crucial to reduce the expression of HSPs, not only to inhibit the protective effect of HSPs on tumor cells and improve tumor cells sensitivity to thermal stimulation, thus improving the efficacy of low-temperature PTT, but also to facilitate the apoptosis of tumor cells. Therefore, researchers have searched for a series of methods to inhibit HSPs, and below we review several strategies to inhibit the production and expression of HSPs and thus achieve low-temperature PTT. Current therapies targeting HSPs utilize four strategies: 1) HSPs (HSP 90 and 70) small molecule inhibitor, 2) 3. Small interfering RNA for HSPs, 3) Starvation therapy for HSPs inhibition, 4) Cytokines inhibition.

## 2 Strategies for heat shock proteins inhibition

### 2.1 Heat shock proteins small molecule inhibitor

#### 2.1.1 Heat shock protein 90 small molecule inhibitors

HSP 90 is a highly conserved ATP-dependent heat stress protein with increased expression during cellular stress and can correct protein misfolding to maintain its activity and reduce cellular damage (Hong et al., 2013; Hoter et al., 2018). Therefore, the inhibition of HSP 90 expression with small-molecule inhibitors which is an important therapeutic idea for low-temperature PTT. Unlike the side effects of nontargeted therapies such as chemotherapeutic drugs, small molecule inhibitors particularly inhibit tumor cell growth by inhibiting certain nucleic acids and proteins associated with tumor growth and metastasis with minimal adverse effects. Moreover, combining HSP 90 small molecule inhibitors with nanomaterials can not only solve the problem of poor targeting but also reduce the adverse effects on other organs and tissues, further improving the therapeutic effect (Zhao et al., 2017).

Geldanamycin, a small molecule inhibitor of ATP-competitive HSPs, which binds to HSP90 and alters the construction of HSP90, inhibiting ATPase activity required for HSP90, thereby preventing HSP90 from binding properly to its client proteins and ultimately leading to the degradation of its client proteins by proteases (Stebbins et al., 1997). 17-Allylamino-17-demethoxygeldanamycin (17-AAG) is a derivative obtained by substituting the 17th methoxy position of geldanamycin with N,N-dimethylethylamine, which has substantially increased biological activity and improved toxicity compared to geldanamycin (Yun et al., 2015; Todurga Seven et al., 2022). Hollow mesoporous organosilica nanocapsules (HMONs) designed by Wu et al., which were loaded with indocyanine green (ICG) and 17-AAG onto HMONs using gemcitabine (Gem) molecules as capping ends, and after correction with polyethylene glycol (NH_2_-PEG), the assembled ICG-17AAG@HMONS-Gem-PEG exhibited pH responsive molecular release properties and glutathione (GSH)-dependent degradation properties, which can precisely control the release of ICG and 17AAG to accomplish low-temperature PTT (Wu et al., 2018). Using a similar application of 17-AAG, Fu et al. designed and synthesized a boron (B)-based multifunctional nanoplatform with NH_2_-PEG-NH_2_ and cRGD (targeting αvβ3 integrins overexpressed in tumor cells), respectively, by “electrostatic adsorption” and “amide reaction” surface modification of B nanosheets (B-PEG-cRGD), which both prolongs the half-life of the platform *in vivo* and enables the platform to specifically target tumor sites and then coloads adriamycin (DOX) and 17-AAG onto B-PEG-cRGD nanosheets to form a multifunctional nanoplatform (DOX-17AAG@B-PEG-cRGD). The NIR laser and an acidic pH environment will accelerate the release of DOX and 17-AAG from the above nanoplatform to achieve low temperature PTT to kill tumor cells (Fu et al., 2020).

**FIGURE 1:**
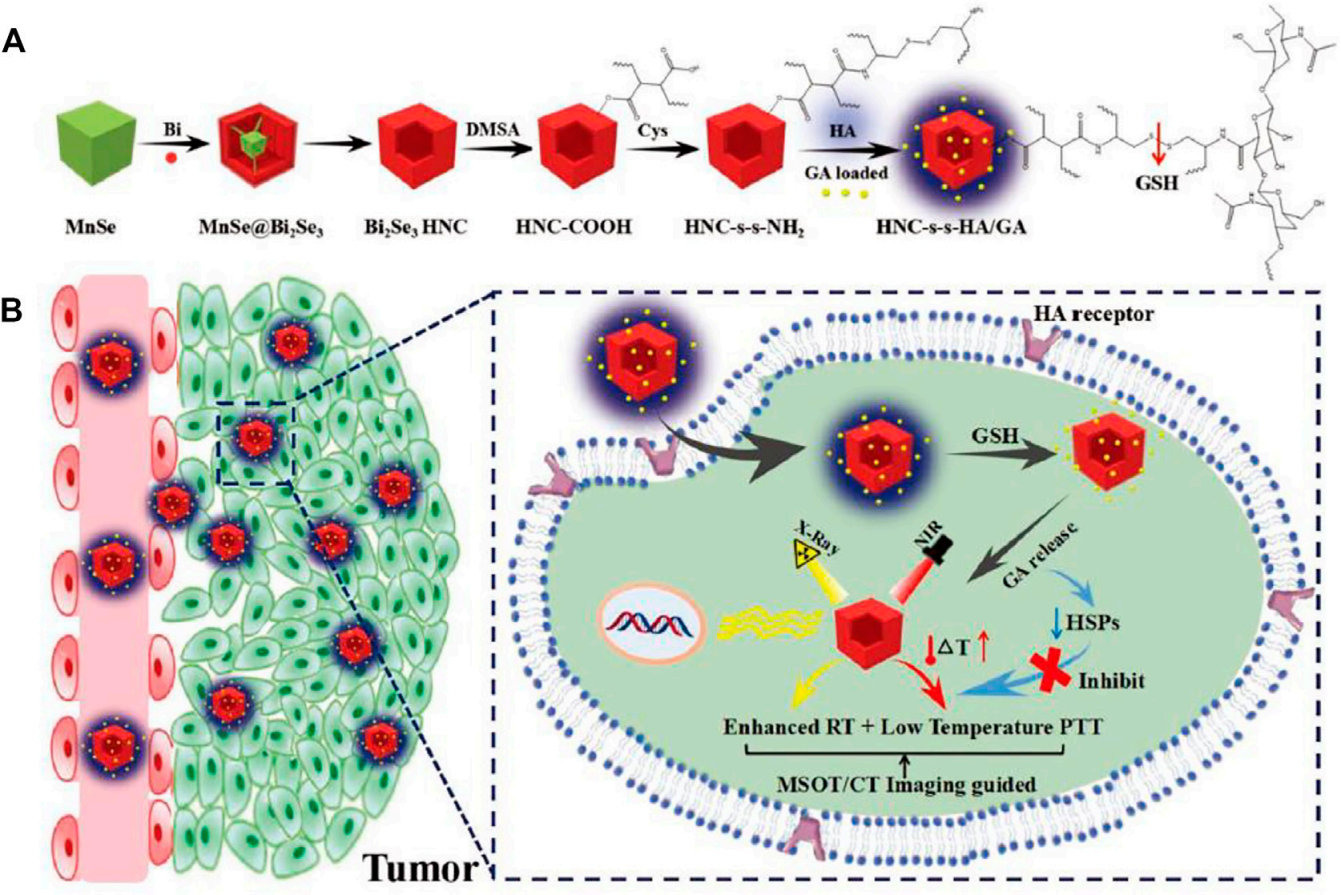
Schematic diagram of **(A)** the construction of Bi2Se3 HNC-s-s-HA/GA (HNC-s-s-HA/GA), **(B)** HNC-s-s-HA/GA provoked low-temperature PTT and boosted RT (Song et al., 2019).

Another natural small molecule inhibitor of HSPs, gambogic acid (GA), suppressed cell proliferation and induced degradation of client proteins of HSPs in cells (Davenport et al., 2011). Bi@ZIF-8 (BZ) nanoparticles were prepared by Li et al. Then, GA was effectively loaded onto BZ nanomaterials by a physical mixing method. Under acidic conditions, the drug release rate increased significantly with increasing temperature (Li J. et al., 2020). Sun et al. synthesized non-toxic, high photothermal conversion hollow mesoporous carbon spheres (HMCS) to form the HMCS-PEG-GA system with GA loading. HMCS-PEG-GA showed good stability in biological media and good photothermal imaging and photoacoustic imaging (PAI) capability. Guided by PAI signals, HMCS-PEG-GA shows great promise for accurate tumor diagnosis and low-temperature PTT (Sun et al., 2020). In addition, Yang et al. combining metal ions Mn^2+^ and ICG together *via* poly-l-histidine-polyethylene glycol (pHis-PEG) to form one-dimensional nanoscale coordination polymers (1D-NCPs).After that, GA was loaded in 1D-NCPs to form finally nanoplatform (Mn-ICG@pHis-PEG/GA), which exhibited effective pH responsiveness after systemic administration, and in low-temperature photothermal conditions, they can then effectively target tumor cells and induce apoptosis to achieve low-temperature PTT (Yang et al., 2017). They not only developed a facile method to prepare 1D-NCPs that can bind to different metal ions, but the nanoplatform is pH-responsive and can improve tumor targeting efficiency and specificity in a more direct manner. Similarly, inhibiting the expression of HSP90 and regulating nanoparticle release and degradation depends on the pH in the TME, thus improving the effectiveness of cryogenic antitumor therapy. Gao et al. self-assembled GA with human serum albumin (HSA) and DC-IR825 (an anabolic dye and photothermal agent) in aqueous solution through simple mixing to form stable HSA/DC-IR825/GA, which then escapes into the cytoplasm by mitochondrial breakage under NIR laser irradiation, releasing GA and blocking high temperature-induced HSP90 overexpression. In addition, HSA/DC-IR825/GA nanoparticles exhibited pH-responsive charge reversal properties, effective tumor site aggregation properties and minor liver deposition effects, which ultimately promoted the low-temperature PTT effect (Gao et al., 2019). Unlike simple material mixing or self-assembly, Song et al. prepared Bi_2_Se_3_ hollow nanocubes (HNCs) *via* moderate cation exchange and Kirkendall effect, and then revised it with hyaluronic acid (HA) based on disulfide bonds (-s-s-), which enabling HNC to target CD44 high expression tumor cells. Finally, GA, as a heat-shock protein inhibitor, was loaded into Bi_2_Se_3_-HNC to form HNC-s-s -HA/GA, which could induce effective tumor cell apoptosis under NIR laser irradiation. In addition, based on the radiosensitizer Bi_2_Se_3_-HNC, the nanoparticles can also be used for depth-independent RT (Figure 1) (Song et al., 2019). This study designed a method to synthesize nanoparticles by chemical bonding linkage to control the effective release of GA for more targeted action on tumor cells, while combining low-temperature PTT with RT to achieve more powerful antitumor therapeutic effects.

Mitochondria-targeted low-temperature PTT is a agreeing therapeutic strategy which maximize antitumor effects and reduce adverse effects. Zhang. et al. synthesized a mitochondria-targeted nanomicelles based on IR780 and BIIB021 (a small molecule inhibitor of HSP90) (Yan et al., 2017). PEG-IR780-BIIB021 nano-micelles have good biocompatibility and biostability, have a high encapsulation rate of BIIB021, can be selectively enriched in mitochondria, and can be released after NIR irradiation. The release of BIIB021 after NIR light irradiation not only reduces cellular tolerance to heat but also decreases mitochondrial membrane potential, which resulting in key endogenous apoptotic factors affection (Cyt-C, Caspase-9, Bcl-2 and Bax), synergistically exerting the effect of low-temperature PTT to strike tumor cells down (Zhang et al., 2021). Deng et al. combined low-temperature PTT with the induction of cellular autophagy to exert a more complete tumor cell killing effect. The tumor-targeting ligand folic acid (FA) was introduced into SNX-2112 (HSP90 small molecule inhibitor) (Liu et al., 2012) loaded graphene oxide (GO) to assemble GO-FA-SNX-2112 nanoparticles. GO has photothermal properties, and with heating from NIR irradiation, the non-covalent interaction between GO and other materials was attenuated, promoting the release of SNX-2112, while GO-FA-SNX-2112 could alter the level of tumors autophagy within 90 s after NIR light irradiation. Thus, combining low-temperature PTT with autophagy resulted in significant antitumor activity (Deng et al., 2020). Epigallocatechin gallate (EGCG) is a extracted from green tea and a natural small molecule inhibitor for HSP90. EGCG has low cytotoxicity, good stability, which can selectively interfere HSP90 activity through multiple cellular signaling pathways (Palermo et al., 2005; Bhat et al., 2014). For real-time diagnosis, Jiang et al. proposed a novel diagnostic method by synthesizing NaLuF4:Nd nanoparticles (Lu:Nd)-coated mesoporous SiO2 (mSiO2) to form a core-shell structure (Lu:Nd@mSiO2). After alkaline conditions, the flowerlike Lu:Nd@NiSiO3 was obtained *via* continuously reacted with the nickel-ammonia complex under high temperature. Then, loading EGCG by “electrostatic adsorption” to form finally product of Lu:Nd@NiS_2_-EGCG for NIR imaging and MRI (Jiang et al., 2019). The above examples of integrating various HSPs small molecule inhibition into the PTT nanoplatform not only provide more insights into the working mechanism of low-temperature PTT but also provide important strategies and ideas for achieving safe and effective low-temperature PTT-induced tumor apoptosis.

#### 2.1.2 Heat shock protein 70 small molecule inhibitor

Heat shock protein 70 (HSP 70) is a potent negative regulator of apoptosis and an important factor in promoting tumor cell growth and survival (Elmallah et al., 2020; Kabakov and Gabai, 2021; Lei et al., 2021; Sharapova et al., 2021); In addition, HSP70 level elevation in tumors was correlated with poor patient prognosis, and HSP70 deficiency speeds up tumor cell death and often selectively sensitizes malignant tumor cells to some chemotherapeutic agents (Ciocca and Calderwood, 2005). Similarly, many researchers have also reduced heat resistance to achieved apoptosis of tumor cells at lower temperatures by inhibiting the expression of HSP70. Therefore, the development of small molecule inhibitors or nanocarriers for inhibiting HSP70 expression will provide another strategy to improve low-temperature PTT.

LY294002 is a small molecule inhibitor that inhibits phosphatidylinositol 3-kinase (PI3K)/Akt activity, glycogen synthase kinase-3 (GSK-3) activity, and then reduces HSP70 expression *via* the above (PI3K/Akt/GSK-3/HSP) signaling pathway (Wigmore et al., 2007). PI3K/Akt is a pivotal kinase which involves in the intracellular antiapoptotic pathways by regulating relevant downstream proteins. Accordingly, PI3K/Akt inhibition can promote apoptosis and inhibit cell growth, proliferation, invasion, and tumor metastasis. However, the inhibitor itself is insoluble in water and exhibits severe toxicity in therapeutic indications, which widely limits its wide application. Therefore, a multifunctional Bi_2_S_3_-Tween 20 nanoplatform loaded with LY294002 was formed by Song et al. The Bi_2_S_3_-Tween 20@LY294002 had good biocompatibility and not only exhibited excellent photothermal efficiency under the action of an 808 nm laser but also rapidly removed the ultrasmall Bi_2_S_3_ nanodots from the body and could be used as a safe drug delivery platform *in vivo* (Song et al., 2018). Similarly, to address a series of side effects of conventional PTT, Ni et al. prepared C6TI/Apo-Tat nanoparticles by cowrapping C6TI (photothermal agent) and Apo (heat shock protein 70 inhibitor) and then coupling them with TAT peptide to improve cell penetration. The results demonstrate that C6TI/Apo-Tat NPs can achieve low temperature PTT by inhibiting HSP70 expression (Ni et al., 2020).

A NIR corresponding nanoplatform combining low-temperature PTT with heat-triggered NO donors (RSNO) was designed by You et al. Specifically, a layer of mesoporous silica (SiO_2_) was coating on high-performance gold nanorods (Au) to obtain multifunctional Au@SiO_2_, which were further coupled with S-nitrosothiols on their surface, and then, low-toxicity, stable 2-phenylethynylsulfonamide (PES) (HSP70 inhibitor) was wrapped into Au@SiO_2_ to reduce the tumor cell heat resistance. Subsequently, polyethylene glycol (PEG) was modified onto Au@SiO_2_ to provide better stability and biocompatibility. When PES/Au@SiO_2_ entered tumor cells, the generated hypothermia under NIR irradiation not only caused apoptosis or necrosis under low temperature with the aid of an HSP70 inhibitor but also promoted NO releasing, which achieving the synergistic effect of low-temperature PTT and gas therapy. *In vitro* and *in vivo* experiments showed that this method has high synergistic efficiency and can effectively inhibit tumor growth (You et al., 2020).

Quercetin is a natural product with excellent antioxidant capacity against free radicals; it also inhibits tumors and exerts anticancer effects; in addition, quercetin is considered to be one of the most potent small molecule HSPs inhibitors, inhibiting the expression of HSP70 by interfering with the HSPs transcription factors phosphorylation. The ketone and hydroxyl groups of quercetin presented three possible metal chelating sites and can bind to various metal ions (De Souza and De Giovani, 2004). In this regard, Yang et al. developed multifunctional Qu-Fe^II^P nanospheres based on the coordination interactions between quercetin, iron (Fe) and polyvinylpyrrolidone (PVP) (Yang et al., 2019). The prepared Qu-Fe^II^P package carries quercetin as the backbone component of the nanodrug, thus avoiding problems of drug loading and release; Meanwhile, the hydroxyl group of quercetin is transformed to ketone during free radical scavenge (Valko et al., 2006). This weakens chelating ability of quercetin with metals and facilitate the decomposition and scavenging of Qu-Fe^II^P nanodrug *in vivo*; meanwhile, ROS scavenging can alleviate the ROS-related inflammatory response. After, free quercetin can continue to exert its inherent anticancer and anti-inflammatory effects (Middleton et al., 2000). This provides us with a widely used nanotherapeutic platform that can combine multiple metal ions while encapsulating multiple small molecule inhibitors of HSPs to construct various nanosystems for low-temperature PTT.

#### 2.1.3 Small molecule inhibitors of heat shock protein 90/70

To inhibit both HSP90 and HSP70 and exert a better effect of inhibiting HSPs, thus further improving the therapeutic effect of low-temperature PTT, researchers applied both HSP90 and HSP70 small molecule inhibitors. Miyagawa, T. et al. Investigated 17-DMAG (HSP90 inhibitor) and quercetin (HSP70 inhibitor) with ferromagnetic particle (FMP)-mediated antitumor effects of low-temperature PTT and found that tumor growth was significantly inhibited (Miyagawa et al., 2014). Instead of applying two small molecule inhibitors of HSPs simultaneously, Tang et al. applied VER-155008, a small molecule inhibitor of both HSP90 and HSP70, to nanomaterials and established a therapeutic system consisting of methoxy-polyethylene-glycol-coated-gold-nanorods (MPEG-AuNR) and VER-155008 micelles (VER-M), which improved the biocompatibility and boosted permeability and retention (EPR) effects of the nanomaterials and reduced toxicity (Tang et al., 2018; Brünnert et al., 2020; Sakai et al., 2021).

### 2.2 Small interfering RNA (siRNA)

SiRNA regulate the expression of HSPs at the gene level through gene silencing. Many problems are encountered during RNA interference processing. First, during blood transport, siRNA cannot be delivered alone due to its negative charge. Secondly, therapeutic siRNA suffers from low cellular delivery efficiency as well as nontargeting. Finally, after siRNA is taken into the cell and then escapes from the nuclear endosome into the cytoplasm, many factors that interfere with RNA are also encountered in the cytoplasm. Finally, when siRNA is taken into the cell and then escapes from the nucleosome to the cytoplasm, many factors that interfere with RNA are encountered in the cytoplasm, such as cleavage by nucleic acid endonucleases and RNA-induced silencing complex (RISC). To overcome these delivery barriers, researchers have designed various nanocarriers to enhance the blood stability of siRNA, cellular uptake of siRNA and escape from the nucleosome (Lee et al., 2012).

**FIGURE 2:**
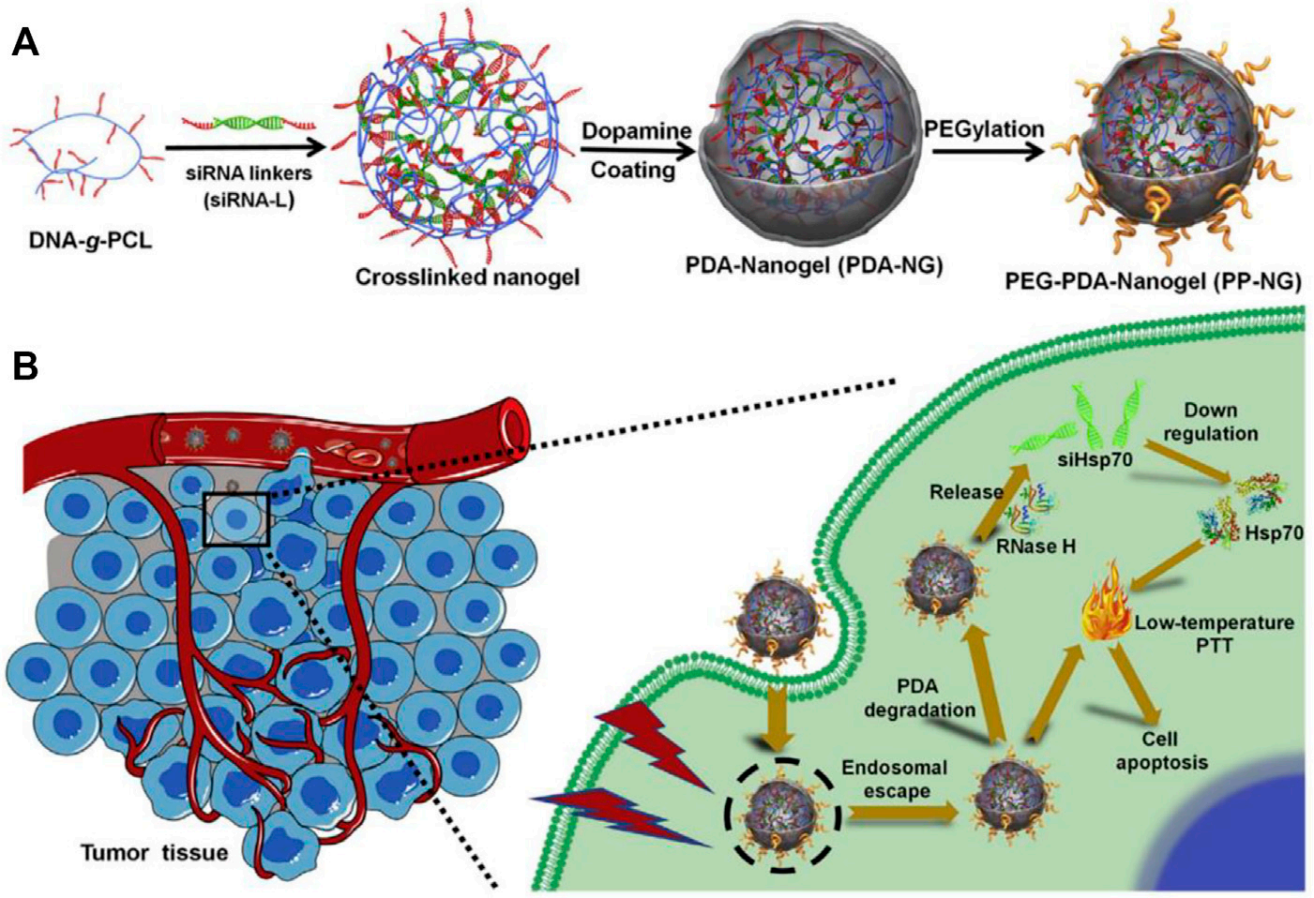
Schematic diagram of **(A)** PDA-encapsulated nucleic acid nanogels and **(B)** mediated low-temperature PTT (Ding et al., 2020).

Wang et al. developed a multifunctional photothermal platform consisting of a gold nanoshell core wrapped around siRNA targeting HSP70. siRNA can specifically detach from the gold surface and achieve *in vivo* lysosomal escape under NIR light irradiation, thereby inhibiting the expression of HSP70(Wang et al., 2017). This strategy cleverly combines local PTT and controlled HSP70 silencing with a simple and easily controlled structure, which has considerable potential for clinical applications. Similarly, inhibiting the expression of HSP70, Ding et al. constructed a HSP70-siRNA worked as crosslinkers to direct the DNA-grafted polycaprolactone (DNA-g-PCL) assemble into nanosized hydrogel (dopamine (PDA)-coated nucleic acid nanogel) through nucleic acid hybridization. After that, the siRNA-embedded nanogels was coated by a thin layer of polydopamine. The assembled siRNA delivery complex had good blood stability, enhanced accumulation at the tumor site, and ablated tumors effectively under low temperature light conditions (Figure 2) (Ding et al., 2020). In response to the lack of specific surface markers to distinguish triplenegative breast cancer (TNBC) from normal cells, Wang. et al. used a layer-by-layer preparation to synthesize a gold nanostar (GNS)/siRNA72/hyaluronic acid (HA) nanosystem to selectively target and sensitize tumor cells to PTT. Despite CD44 is a good marker for triple-negative breast cancer, it is also highly expressed in other normal tissues, such as human breast epithelium (Jordan et al., 2015). Therefore, HA alone cannot completely distinguish triple-negative breast cancer from adjacent normal breast tissue. The experimental design of GNS/siHSP72/HA, which specifically introduces siHSP72 into CD44-positive cells, particularly sensitizes triple-negative breast cancer cells by downregulating HSP72 overexpression in CD44-positive cells and improves the effect of PTT on TNBC with lowest damage to healthy cells. By targeting CD44 and subsequently removing of HSP72, the therapeutic window of PTT was successfully narrowed from irradiating all cells to only CD44-positive cells and TNBC cells (Wang S. et al., 2016). This undoubtedly reduced damage to normal cells and improved targeting of abnormal cells.

BAG3 protein is belong to BAG cochaperone protein family, which is the only member of the family induced by stressful stimuli, mainly through activation of heat shock factor 1 (HSF1) on the promoter of the BAG3 gene, and plays a cytoprotective role in preventing cell death and an important role in heat resistance (Rosati et al., 2011; De Marco et al., 2018; Marzullo et al., 2020). Wang et al. modified gold nanorods (GNRs) with positive dots on the surface and then bound to negative ionized siRNA targeting BAG3 by “electrostatic adsorption” to form GNRs-siRNA nanocomplexes. The nanocomplex could inhibit high temperature-induced BAG3 expression at the mRNA and protein levels and produced stable NIR-activated photothermal properties (Wang B. et al., 2016).

Despite the considerable research potential of siRNA drugs, they have not been strongly promoted in scientific experiments and clinical drug applications due to problems such as poor blood stability and nontargeted effects. To solve these critical problems, not only the structure of siRNA needs to be optimized, but specialized carrier transport systems also need to be developed. At present, researchers have designed many strategies to optimize the structure and transport carriers of siRNA.For example, siRNA can be assembled by simple chemical ligation, complementary base pairing and interaction forces with nanocarriers to form stable complexes, which can not only compensate for the defects of easy degradation of a single siRNA but also improve the intracellular delivery. New siRNA transport carriers were designed with better siRNA delivery properties to avoid other undesirable reactions while maintaining the inherent biological activity. Furthermore, by introducing a cleavable chemical bond between siRNA and the carrier, the siRNA and the nanocarrier can be easily dissociated from each other and the original siRNA can be released to function in a specific environment. Of course, we still need to perform rigorous biological characterization and experimental validation of the newly fabricated siRNA-based structures and carriers to identify and resolve their possible adverse reactions.

### 2.3 Starvation therapy

Although there are numerous small molecule inhibitors of HSPs, their effect is delayed because they only act on the stimulated HSPs that are already presentand do not prevent their production in advance, so they do not inhibit the effect before treatment. Moreover, tumor cells can overexpress multiple HSPs, but one siRNA and small molecule inhibitors of HSPs may have a single effect and can only impair the expression of one heat shock protein. Therefore, some researchers have considered further inhibiting the production of HSPs at the source. Tumor “starvation therapy” is an emerging approach to inhibit tumor growth by cutting off nutrient supply, which is usually achieved through vascular embolization (Jing et al., 2018). In addition, rapid tumor cells proliferation requires a large amount of vitality to survive and grow, causing tumor cells to absorb more glucose than normal cells and become vulnerable to changes in glucose concentration.

Glucose oxidase (GOx) is the most commonly used biocatalyst for regulating tumor glucose metabolism; Gox converts glucose to gluconate and toxic H_2_O_2_ and inhibits the production of ATP by promoting glucose consumption, which is the main energy donor of the body and is important for both cell growth and protein synthesis. In addition, tumor hypoxia is a great challenge in tumor therapy due to negative feedback of various antitumor treatments. Zhou. et al. designed a composite nanosystem that targets tumor cells and has redox-responsive properties. The nanosystem loads GOx onto porous hollow Prussian blue nanoparticles (PHPBNs) and coats hyaluronic acid (HA) on its surface *via* disulfide bonds (-s-s-). The PHPBN can catalyze the breakdown of H_2_O_2_ to O_2_ in tumor cells to enhance hypoxia-inhibited GOx-mediated therapy. Furthermore, PHPBNs and GOx enhance intracellular glucose consumption and reduce ATP supply, which further reduce the resistance to PHPBN-mediated low-temperature PTT (Zhou et al., 2018). Using the same application of GOx, Song. et al. designed a nanoparticle using PDA as the core and hollow silica (PDA@hm) as the shell to carry the autophagy inhibitor drug chloroquine (CQ) and GOx to form PDA@hm@CQ@GOx. PDA@hm@CQ@GOx can effectively mediates low-temperature PTT of tumors through starvation effect. Moreover, Gao. et al. rationally designed a multifunctional temperature-sensitive liposome, and constructed temperature-sensitive phase transition liposomes through lipid molecules DPPC and DSPE-PEG2000 to compress GOx, ICG, and GA. The formed GOIGLs could achieve thermally responsive drug delivery at tumor sites and hypothermic hyperthermia (Gao et al., 2020).

**FIGURE 3:**
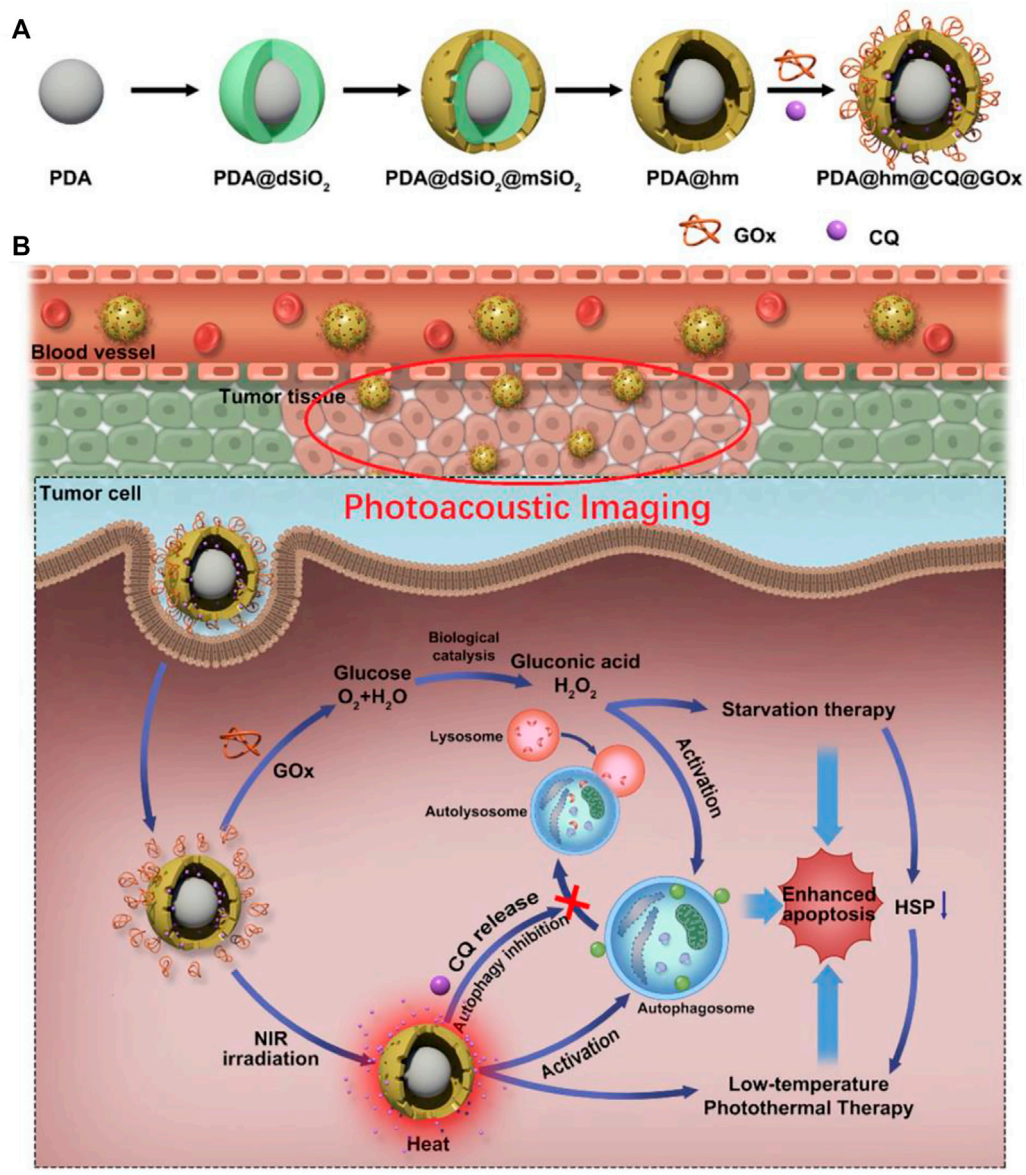
Schematic diagram of **(A)** the construction of PDA@Hm@CQ@GOx and **(B)** the enhancement of low-temperature PTT through inhibition of autophagy and energy metabolism (Shao et al., 2020).

Moreover, the combined catabolism of glucose to inhibit ATP production and inhibition of autophagy showed the good effect of PDA@hm@CQ@GOx for boosting low-temperature PTT (Figure 3). Firstly, PDA@hm was formed *via* a four-step process. Then, the autophagy inhibitor CQ was loaded in PDA@hm, which further combined with glucose consumer GOx to form PDA@hm@CQ@GOx ( corona-like structure) (Shao et al., 2020). Cao. et al. designed a nanoreactor Fe-PDAP/GOx/ICG for enhancing low-temperature PTT using a combination of mechanisms. Glucose is broken down by GOx to gluconic acid and H_2_O_2_, which is converted to O_2_ to promote oxygenation, thereby enhancing O_2_-dependent photodynamic therapy (PDT). Results showed that the nanoreactor not only reduced tumor cell heat resistance, but also enhanced intracellular ROS production and cytotoxicity in tumor cells, successfully achieving a multimechanism combination to induce low-temperature PTT (Cao et al., 2020). Unlike GOx, which promotes glucose consumption, lonidamine (LND) is an antitumor drug that reduces ATP production indirectly by regulating the secretion of hexokinase, a key biochemical process in energy production such as glycolysis and mitochondrial respiration (Nath et al., 2016). From this, Wang. et al. designed tumor cell membrane-encapsulated nanoparticles targeting tumor cell mitochondria. ZnGlu-PB nanoparticles (PG NPs) were synthesized by encapsulating the photothermal agent Prussian blue nanoparticles (PB NPs) with zinc glutamate (ZnGlu) and then coupling the LND molecule to the mitochondrial targeting group TPP (denoted TLND) (PGTL NPs) to increase the inhibitory effect on ATP production. Finally, HepG_2_ cell membranes were wrapped around PGTL to form HmPGT NPs, which considerably prolonged the *in vivo* circulation time and interfered with ATP production to improve treatment effect of low-temperature PTT to tumor cells (Wang P. et al., 2021). The production of ATP is dependent on glucose enzymes, which to a large extent requires the help of glucose transporters (Gluts) to transfer glucose from extracellular to intracellular. The typical representative is Glut1, which is widely express in tumor cells (Macheda et al., 2005). Chen et al. developed a GNR/HA-DC nanosystem by coupling diclofenac (DC), a highly selective inhibitor of Glut1, and hyaluronic acid (HA) and further modifying them on gold nanorods (GNRs). Upon reaching the tumor site, tumor cells overexpressing hyaluronidase (HAase) can cleave the nanosystem and trigger DC release, and the released DCs induce downregulation of Glut1, inhibiting glucose transport, metabolism and ATP-dependent HSPs synthesis for the purpose of low temperature PTT (Chen et al., 2017).

### 2.4 Cytokines (TNF-α, IL-2 and IFN-γ *etc.*)

Cytokines secreted by immune cells (such as TNF-α, IL-2, IFN-γ, *etc.*) play a key role in antitumor immune effect. On the one hand, cytokines can promote tumor apoptosis through enhancing the expression of apoptosis-related proteins (Bax, Caspase 3 and Caspase8), reduce the expression of apoptosis-inhibiting proteins (Bcl-2, Bcl-xL), and promote tumor apoptosis. TNF-αbinds to two receptors, TNF-R1 and TNF-R2, leading to the recruitment of signal transducers and the activation of at least three distinct effectors through complex signalling cascades and networks. These factors then lead to the activation of caspases and proinflammatory transcription factors, AP-1 and NF-κB, trigger apoptosis in target cells. According to Ravichandran et al., apoptotic cell metabolic secretions positively regulate neighbouring cells within tissues. The gene expression of HSPs plays an anti-inflammatory role in the inflammatory environment *in vivo* to stabilize the stress environment (Baud and Karin, 2001; Green, 2020), thereby reducing the defensive overexpression of HSPs in tumor cells. On the other hand, cytokines can inhibit the expression of HSPs families (such as HSP27, HSP70, HSP90, HSP105, and HSF1, etc.) in tumor cells and enhance the sensitivity of tumor cells to heat stimulation (Schett et al., 2003; Sun Z. et al., 2021). For example, TNF-α can reduce amount of HSP27 Ser78 phosphorylation through the activation of PKC, thereby reducing the level of HSP27 in PBMCs (peripheral blood mononuclear cells) (Niwa et al., 2006) and attenuating the tolerance of target cells to thermal stimulation.

**FIGURE 4:**
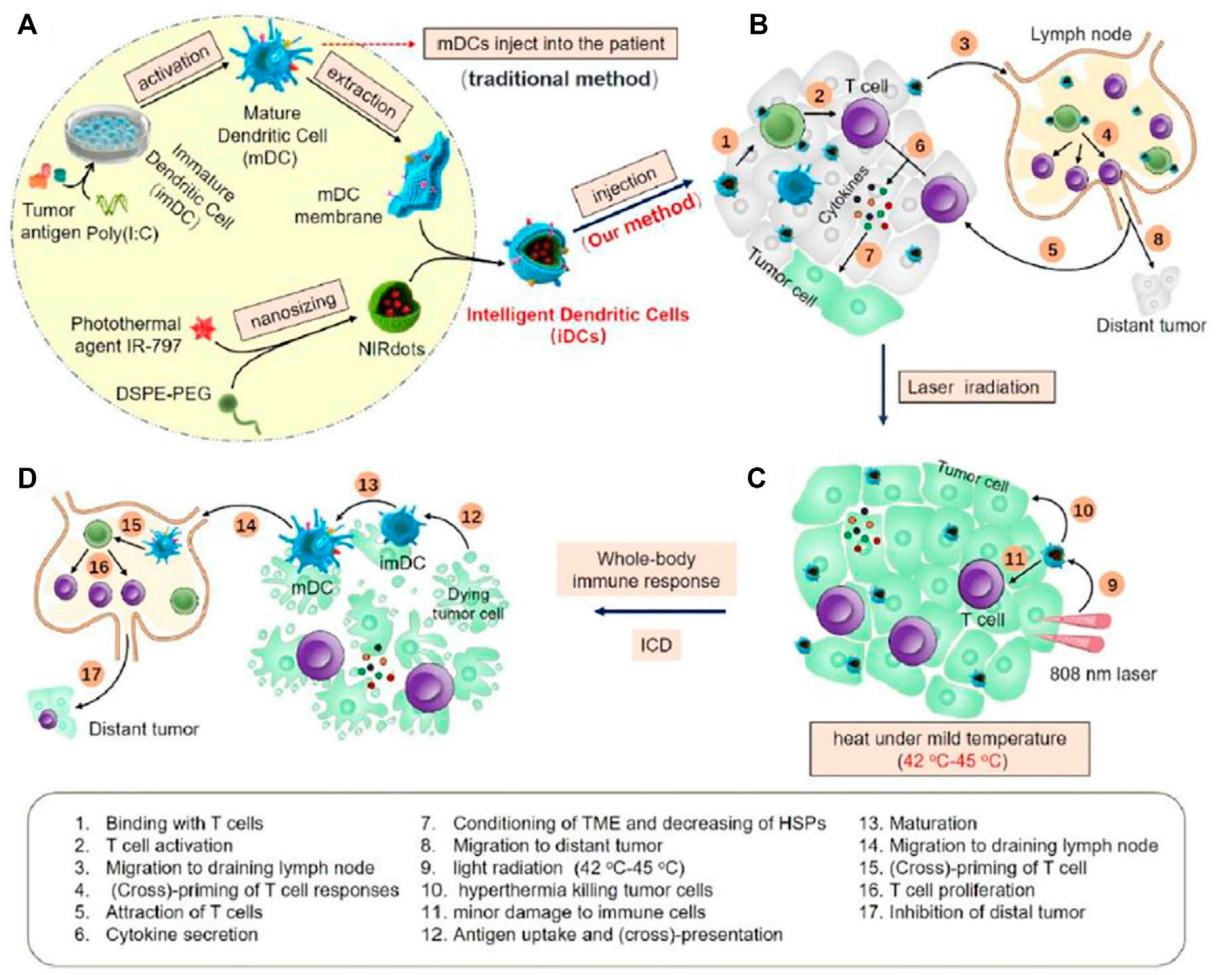
The schematic of iDCs activating T cells and mediating hypothermic PTT. **(A)** The construction of iDCs. **(B)** iDCs injected intratumorally to primary tumor. **(C)** iDCs enhanced low temperature PTT. **(D)** iDCs induced synergistic antitumor effect (Sun Z. et al., 2021).

During tumorigenesis, the expression of tumor necrosis factor receptor-related factor 1 antibody (TNF-R1) on the cell membrane of tumor vascular endothelial cells and tumor cells is upregulated (Sun Z. et al., 2021). In the study of Schett. et al., after antibody (TNF-R1) and protein phosphatase-mediated inhibition of heat shock transcription factor 1 (HSF1) phosphorylation, TNF-a transiently downregulates the HSP70 stress response, resulting in increased cell susceptibility to apoptosis (Sun Z. et al., 2021). However, the immunosuppressive tumor microenvironment (TME) promotes tolerogenic dendritic cells (DCs) generation, the stimulation of CD4^+^ T-cell and CD8^+^ T-cell proliferation and activation is attenuated. Therefore, the design of engineered DCs has considerable potential to promote T-cell killing of tumor cells. Sun et al. designed an artificial DC (iDC) that consists of a nanoparticle core loaded with a photothermal agent (IR-797) and an outer coating of a mature DC film. NIRdots were firstly formulated through a nanoprecipitation procedure. Then, iDCs were further formed by coating mature DC cell membranes on NIRdots using an extruding method. The ability of DC cells to present antigens and activate T cells was retained by the iDCs on the surface of DC cell membrane (Figure 4). T cells activated by iDCs can produce cytokines which inhibit the expression of HSPs (HSP27, HSP70, HSP90, HSP105 and HSF1), as well as promoting tumor apoptosis and synergistically amplifying the antitumor effect of low temperature photothermal treatment (42–45°C). Immune cells and apoptotic tumor cells can induce immunogenic cell death, initiating a cycle of tumor immunity (Sun Z. et al., 2021).

In summary, we have reviewed several ways that researchers have accomplished low temperature PTT by inhibiting HSPs expression in recent years, as summarized in Table 1.

**TABLE 1:** The summary of different inhibitory modes for HSPs in low temperature PTT.

Modalities of inhibition of heat shock proteins (HSPs) expression	Mechanism	Inhibitors	References
HSPs small molecule inhibitor	Inhibition of HSP90	17-AAG	(Wu et al., 2018; Fu et al., 2020)
		Gambogic acid (GA)	(Yang et al., 2017; Gao et al., 2019; Song et al., 2019; Li et al., 2020a; Sun et al., 2020)
		BIIB021	Zhang et al. (2021)
		SNX-2112	Deng et al. (2020)
		Epigallocatechin gallate (EGCG)	Jiang et al. (2019)
	Inhibition of HSP70	LY294002	Song et al. (2018)
		Apo	Ni et al. (2020)
		2-phenylethynylsulfonamide (PES)	You et al. (2020)
		Quercetin	Valko et al. (2006)
	Inhibition of HSP90/70	17-DMAG and Quercetin	Miyagawa et al. (2014)
		VER-155008	Tang et al. (2018)
siRNA	Inhibition of HSP70	si-Hsp70	(Wang et al., 2017; Ding et al., 2020)
	Inhibition of HSP72	si-Hsp72	Wang et al. (2016b)
	Inhibition of BAG3 expression	si-BAG3	Wang et al. (2016a)
Starvation therapy	Promotes glucose consumption, inhibits ATP production, and reduces heat shock protein energy supply	Glucose oxidase (Gox)	(Zhou et al., 2018; Shao et al., 2020)
	Inhibition of key biochemical processes such as glycolysis and mitochondrial respiration for energy production	Lonidamine (LND)	Wang et al. (2021b)
	Highly selective inhibitor of Gluts1, inhibiting the transfer of glucose from extracellular to intracellular	Diclofenac (DC)	Chen et al. (2017)
Cytokines	Inhibition of HSP27, 70, 90, 105 and heat shock transcription factor 1 (HSF1)	TNF-α, IL-2 and IFN-γ *etc.*	Sun et al. (2021b)

## 3 Summary and outlook

PTT is a promising tool for using lasers to treat early-stage tumors or other types of disease. A good photothermal conversion agent can improve the selective heating of the target area. Molecular motion inside matter can generate heat. After decades of development, exciting progress has been made in all aspects. Several models of biological heat transfer which relied on Fourier and non-Fourier effects have been explored. Due to special structures and properties of biological tissues, and many other factors, the effects of metabolic heat production, blood flow, local thermal imbalances, time delays have been considered in different models. In addition, numerical models of radiative transport in biological tissues consider the complex optical organization and photothermal agent interactions (Ren et al., 2022). As an emerging treatment method, PTT has the advantages of strong controllability, remarkable curative effect, and small toxic/side effects, and which has been widely used in cancer research. Generally, most researches focus on PTT at high temperature (>50°C). Although high temperature PTT has good effect, which will also inevitably cause irreversible damage to normal tissues around the tumor. Therefore, low-temperature PTT has developed very rapidly, especially in the application of nanomaterials mediated low-temperature PTT.

In recent years, low-temperature PTT with nanomaterials has attracted increasing attention as an efficient, noninvasive and highly targeted therapeutic modality and has shown considerable potential in solving some problems in the biomedical field; For example, the size and physicochemical properties of nanomaterials make it easier to penetrate biological membranes and enable nanoparticles to specifically target tumor cells by integrating some tumor-targeting molecules or wrapping homologous cell membranes. In addition, nanomaterials have some remarkable properties such as pH responsiveness, organelle targeting, *etc.*, which enable tumor cells to accumulate at the tumor site through the EPR effect. In addition, nanomaterials have some remarkable properties, such as pH responsiveness and organelle targeting, which can enhance the anti-tumor therapeutic effect by significantly increasing the ability of tumor cells to take up and accumulate drugs through low temperature PTT.

In this paper, we review the progress of nanomaterial-mediated low-temperature PTT mainly in terms of inhibiting the expression of HSPs. First, given that HSPs are the main factor in tumor cell heat resistance and play an important role in assisting tumor cells escape apoptosis-mediated death, the use of small molecule inhibitors of heat shock proteins (such as 17-AAG, GA, BIIB021, SNX-2112, EGCG, LY294002, Apo, 17-DMAG, quercetin, VER- 155008, *etc.*) and siRNAs can not only make tumor cells more susceptible to low-temperature PTT but also promote apoptosis; In addition, because HSPs synthesis and biological functions require energy, fundamental inhibition of HSPs production can be achieved by reducing or depleting nutrients or blocking nutrient supply. Second, the combination with autophagy activation/inhibition therapies can further improve the effectiveness of tumor clearance. Third, as many organelles play integral role in maintaining intracellular homeostasis, are susceptible to high temperatures, and combining organelle-targeting strategies with low-temperature PTT, which can also further improve the effectiveness of killing tumor cells. Finally, synergistic treatment combining low-temperature PTT with other therapeutic modalities (e.g., chemotherapy, immunotherapy, PDT, RT, *etc.*) will further improve the efficacy of low-temperature PTT: high temperature can increase blood flow, which can enhance the transport and accumulation of chemotherapeutic drugs and radiosensitizers in tumor cells; Other therapeutic can make cancer cells more susceptible to low-temperature PTT and exert better therapeutic effects; In addition, studies have also confirmed that high temperature can improve the hypoxic environment at the tumor site, providing the basic conditions for oxygen-dependent therapeutic modalities such as PDT. Therefore, nanotechnology is crucial for achieving synergistic therapeutic effects, and using diverse modification and synthesis methods, various drugs, materials and technological approaches with different therapeutic effects can be assembled and combined to exert better anti-tumor effects while achieving higher efficacy and fewer side effects.

Although there have been many innovative studies on low-temperature PTT, there are still some issues that we must address. First, although numerous nanocarriers have been developed for low-temperature PTT and have shown satisfactory results, such as efficient drug encapsulation rate and loading capacity, low toxicity to normal cells, and fine biocompatibility, more effective nanocarriers needs to be discovered. First, to improve the delivery capacity, enhance biodegradability, and prolong blood circulation time and stability, more studies should carry out in order to achieve low-temperature PTT. For example, a nanoplatform to target organelles other than mitochondria and lysosomes could be designed, and the effect of acting on different organelles to optimize the targeting of organelles for low-temperature PTT should be compared. In addition, novel therapeutic modalities including iron death, transgenic technology, and gene editing technology, can also be further explored in relation to low-temperature PTT. More importantly, we should identify more detailed mechanisms of action of these synergistic treatments develop more effective combinations and sequences of applications. Finally, there are some reports on low-temperature PTT for other diseases, such as endocrine system diseases, neurological diseases and cardiovascular system diseases, which can further expand the research. Overall, the translation of nanomedicine carriers into real clinical applications still requires a lot of study and the joint efforts of more researchers.
